# Development and Validation of Non-Integrative, Self-Limited, and Replicating Minicircles for Safe Reporter Gene Imaging of Cell-Based Therapies

**DOI:** 10.1371/journal.pone.0073138

**Published:** 2013-08-28

**Authors:** John A. Ronald, Lorena Cusso, Hui-Yen Chuang, Xinrui Yan, Anca Dragulescu-Andrasi, Sanjiv Sam Gambhir

**Affiliations:** 1 Molecular Imaging Program at Stanford, Stanford University, Stanford, California, United States of America; 2 Department of Radiology, Stanford University, Stanford, California, United States of America; 3 Departamento de Bioingeniería e Ingeniería Aeroespacial, Universidad Carlos III de Madrid, Instituto de Investigación Sanitaria Gregorio Marañón, CIBERSAM, Madrid, Spain; 4 National Yang-Ming University, Taipei, Taiwan; Wayne State University, United States Of America

## Abstract

Reporter gene (RG) imaging of cell-based therapies provides a direct readout of therapeutic efficacy by assessing the fate of implanted cells. To permit long-term cellular imaging, RGs are traditionally required to be integrated into the cellular genome. This poses a potential safety risk and regulatory bottleneck for clinical translation as integration can lead to cellular transformation. To address this issue, we have developed non-integrative, replicating minicircles (MCs) as an alternative platform for safer monitoring of cells in living subjects. We developed both plasmids and minicircles containing the scaffold/matrix attachment regions (S/MAR) of the human interferon-beta gene, driven by the CMV promoter, and expressing the bioluminescence RG firefly luciferase. Constructs were transfected into breast cancer cells, and expanded S/MAR minicircle clones showed luciferase signal for greater than 3 months in culture and minicircles remained as episomes. Importantly, luciferase activity in clonal populations was slowly lost over time and this corresponded to a loss of episome, providing a way to reversibly label cells. To monitor cell proliferation *in vivo*, 1.5×10^6^ cells carrying the S/MAR minicircle were implanted subcutaneously into mice (n = 5) and as tumors developed significantly more bioluminescence signal was noted at day 35 and 43 compared to day 7 post-implant (p<0.05). To our knowledge, this is the first work examining the use of episomal, self-limited, replicating minicircles to track the proliferation of cells using non-invasive imaging in living subjects. Continued development of S/MAR minicircles will provide a broadly applicable vector platform amenable with any of the numerous RG technologies available to allow therapeutic cell fate to be assessed in individual patients, and to achieve this without the need to manipulate the cell's genome so that safety concerns are minimized. This will lead to safe tools to assess treatment response at earlier time points and improve the precision of cell-based therapies.

## Introduction

Cell-based therapies have emerged as novel therapeutics for the treatment of a variety of diseases including cancer, cardiovascular and neurodegenerative diseases. Promising examples include adoptive immunotherapy for cancer treatment [1] and stem cell therapy for the regeneration of ischemic heart disease [2]. Unfortunately, traditional readouts of treatment success are often indirect (e.g., tumor shrinkage) and only assessable long after cell delivery, making timely adjustment of treatment course difficult [3]. Direct, repeatable monitoring of the fate of delivered cells will allow therapeutic efficacy to be assessed at earlier time points, improve the ability to identify responders and non-responders, and overall, allow more precise cell-based therapies to become a reality.

Non-invasive imaging of therapeutic cells is the most promising approach for monitoring cell fate. In particular, imaging strategies using reporter genes (RGs) can provide information of the location(s), number, viability and differentiation status of delivered cells. Many RG technologies have been developed in the last few decades for imaging modalities such as fluorescence (FL) [4] and bioluminescence (BLI) imaging [5], [6], magnetic resonance imaging (MRI) [7], positron emission tomography (PET) [8], and for emerging technologies such as photoacoustic imaging [9]. Despite these tremendous advances only a single study has translated one of these RG technologies into tracking of therapeutic cells in patients [10]. One of the main reasons for this is potential safety concerns regarding the genetic modification of cells using integrating vectors that have the potential to cause insertional mutagenesis and malignant transformation of cells. This concern has been known for a long time but has become a reality ever since 2 male patients treated with integrating retroviruses developed T-cell acute leukemia-like syndrome during a gene therapy trial to treat X-linked severe combined immunodeficiency disease (SCID-X1) [11], [12]. In order to avoid this serious issue and safely track proliferating cells in humans, non-integrative (episomal) vector platforms with autonomous replicative capability would have significant potential.

In the past decade several groups have reported the development of non-viral vectors containing the human interferon-beta (hIFN-ß) scaffold/matrix attachment region (S/MAR) [13]–[16]. Once introduced into cells in culture, S/MAR vectors remain episomal and can recruit host replication machinery to promote vector replication once per cell cycle [17]. Over time, optimization of S/MAR vectors has been achieved with several groups showing that removal of prokaryotic sequences to generate S/MAR minicircles (MCs) allows dividing cells to be labeled for several generations without the need for antibiotic selection and with minimal integration events [14]–[16]. While the episomal nature and replicative ability of S/MAR constructs has been established in both cultured cells [13]–[16] and transgenic pig fetuses [18], the ability of these constructs to replicate when delivered to tissues *in vivo* (without some form of selection advantage [19]) has been difficult [16]. Despite these challenges for their use as gene therapy vehicles, we were inspired by the results shown in cultured cells using S/MAR MCs and hypothesized that this technology could be extended beyond the culture dish and be used to safely track transplanted RG labeled cells in living subjects.

Therefore, our objectives in this study were: 1) to develop S/MAR MCs that expressed a bioluminescence RG to allow dividing cells to be imaged both in culture and in living mice; 2) to show that these constructs would express transgenes and remain episomal for extended periods of time in culture; and 3) to investigate whether RG labeled cultured cells could be transplanted into animals and their proliferation and viability could be monitored over time with non-invasive imaging. To our knowledge, this is the first work demonstrating the ability to track cells in living subjects using replicating episomal MCs and lays the foundation for future vectors expressing clinically relevant RGs for imaging modalities such as PET or MRI, so that therapeutic cells can be tracked in patients.

## Materials and Methods

### Ethics Statement

The Administrative Panel on Laboratory Animal Care at Stanford University approved all animal experiments and all efforts were made to minimize animal suffering.

### Vector Construction

**Figure 1:**
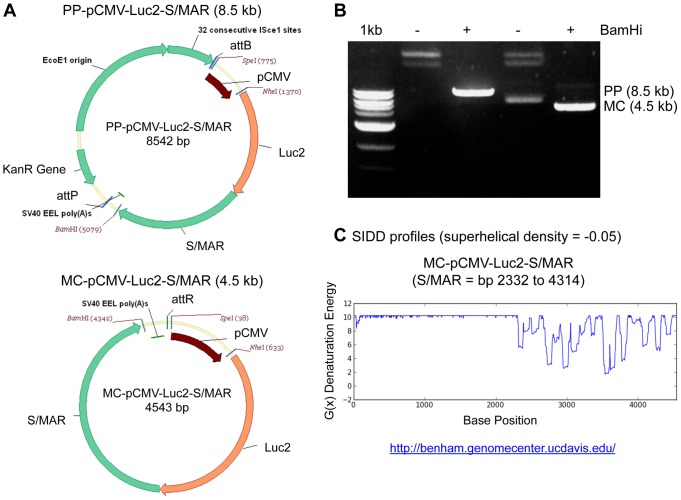
Design and construction of self-replicating minicircle constructs. A) Vector maps of both parental plasmid (PP; top) and minicircle (MC; bottom) versions of pCMV-Luc2-S/MAR. B) Agarose gel electrophoresis analysis confirming the ability to generate both PP (8.5 kb) and MC (4.5 kb). C) Stress induced duplex destabilization (SIDD) profile of MC-pCMV-Luc2-S/MAR at a standard superhelical density of −0.05. Note the regular low denaturation energies (G(x)) between base pairs 2332 and 4314 corresponding to the location of the S/MAR motif.

The construct pEPI-eGFP was kindly provided by Dr. Hans Lipps [18]. This plasmid is driven by the CMV promoter (pCMV), expresses enhanced green fluorescent protein (eGFP), and contains the S/MAR region from the human IFN-ß gene (∼2.0 kb) directly downstream of eGFP. We replaced eGFP with the codon-optimized bioluminescence reporter gene firefly luciferase (Luc2) to generate pEPI-Luc2. Next, to generate both parental plasmids (PP) and MCs we used the system described by Kay et al [20] (System Biosciences, Mountain View, CA). Briefly, we subcloned the pCMV-Luc2-S/MAR transcription unit out of pEPI-Luc2 and into the MN-100 PP backbone (System Biosciences, Mountain View, CA) containing an SV40 polyA sequence to generate PP-pCMV-Luc2-S/MAR (Figure 1A - top). Both PP-pCMV-Luc2-S/MAR (PP) and MC-pCMV-Luc2-S/MAR (Figure 1A - bottom) were amplified and purified according to the protocol outlined in Kay et al [20] and the supplier's instructions (System Biosciences, Mountain View, CA). Briefly, ZYCY10P3S2T E. coli were transformed with the PP, colonies were picked and E. coli were grown overnight in TB broth. To generate MCs, site-specific recombination via expression of phiC31 integrase was initiated by addition of equal volume of LB broth containing 0.001% L-arabinose and 16 mL NaOH, and cultures were grown for an additional 5.5 hours at 30°C. For the PP, the cells were grown in the same media without L-arabinose supplementation. Endotoxin-free mega kits (Qiagen, Valencia, CA) were used to purify both PP and MC.

### Cell Transfection and BLI in Cell Culture

MDA-MB-231 human breast cancer cells (ATCC, Manassas, VA) were grown in DMEM high glucose medium (Gibco, Carlsbad, CA) supplemented with 10% Fetal Bovine Serum (FBS), and 1% Penicillin/Streptomycin (P/S) solution. For transfection, 2.5×10^4^ cells were plated in 24-well plates and transfected with both PP and MC at equal mass (1 µg) using a linear polyethylenimine transfection agent (jetPEI, Polyplus transfection, Illkirch, France) according to the manufacturer's instructions. After reaching confluency, cells were transferred to 10 cm plates and bioluminescence imaging was performed to detect firefly luciferase (Fluc) activity using a Xenogen IVIS 50 imaging system (Caliper Life Sciences, Waltham, MA) 5 minutes after addition of D-Luciferin (0.03 mg/ml) at 37°C.

### Luminometer Assay

Cells were lysed in 1×Passive lysis buffer (Promega, Sunnyvale, CA) for 15 minutes on ice and lysate was centrifuged at 14,000 rpm for 5 minutes at 4°C. Supernatant was collected and Fluc activity was determined using 10 µL lysate after addition of 100 µL LAR-II substrate (Promega, Sunnyvale, CA) in a TD 20/20 luminometer (Turner Designs, Sunnyvale, CA). An integration time of 10 seconds was used for all measurements recorded as relative light units (RLU). The protein content (µg) of tissue lysates was determined using a Pierce 660 nm Protein Assay system (Thermo Scientific, Rockford, IL) in a BioTek Synergy 4 microplate reader (BioTek Instruments, Winooski, VT). Normalized Fluc activity is reported as RLU per µg of protein.

### Southern blot analysis

Total DNA was isolated from MDA-MB-231 cells using the DNeasy Blood and Tissue Kit according the manufacturer's instructions (Qiagen, Valencia, CA). DNA concentration was quantified using a Nanodrop 1000 spectrophotometer (Thermo Scientific, Wilmington, DE). Total DNA was digested with a single cutting restriction enzyme (EcoRI), and 40 µg was separated on a 0.7% agarose gel (30 V, 20 mA for 7 hours). DNA was then blotted overnight onto Amersham Hybond-N+ paper according to the manufacturer's instruction (GE Healthcare, Buckinghamshire, UK). Finally, a 400 bp Luc2 probe was labeled with alkaline phosphatase using the Amersham Gene Images AlkPhos Direct Labelling and Detection System (GE Healthcare, Buckinghamshire, UK), and chemiluminescent signal detection was performed as per the manufacturer's instructions.

### Tumor Development and *In Vivo* BLI

To perform *in vivo* imaging of cell proliferation, 1.5×10^6^ cells from a S/MAR MC clonal population (clone 3–7; 61 days post-transfection) were implanted subcutaneously into the right flank of Nu/Nu mice (n = 5). BLI was performed on days 7, 20, 28, 35, and 43 post-implantation using a Xenogen IVIS Spectrum imaging system (Caliper Life Sciences, Waltham, MA). Each mouse was injected intraperitoneally with 100 µL of D-Luciferin (30 mg/mL) and a series of images was collected between 5 and 20 minutes post-injection. A region of interest was drawn over the tumor in each image and the peak average radiance (photons/sec/cm^2^/steradian) within the imaging period was measured.

### Statistics

To compare normalized Fluc activity in cultures at days 64 and 121 we performed a paired two-tailed t-test. To compare average radiance measurements taken over time we performed a repeated measures analysis of variance followed by a Tukey's post-hoc test. A nominal p-value less than 0.05 was considered to be significant.

## Results

### S/MAR MCs can label cells with RGs for extended periods of time in culture and remain episomal

We developed both parental plasmids (PPs) and minicircles (MCs) driven by the CMV promoter, expressing a firefly luciferase gene (Luc2), and containing the hIFN-ß S/MAR (pCMV-Luc2-S/MAR; Figure 1A). The PP was approximately 8.5 kb in size, whereas after removal of prokaryotic components the MC was about 4.5 kb (Figure 1B). As previously described [16], the stress induced duplex destabilization (SIDD) profile of the MC (at a superhelical density of -0.05) revealed regular intervals within the S/MAR motif with low denaturation energy (G(x)), demonstrating a high propensity for this region to undergo strand separation (http://benham.genomecenter.ucdavis.edu/; Figure 1C). The PP also showed a similar pattern within the S/MAR region (data not shown).

**Figure 2:**
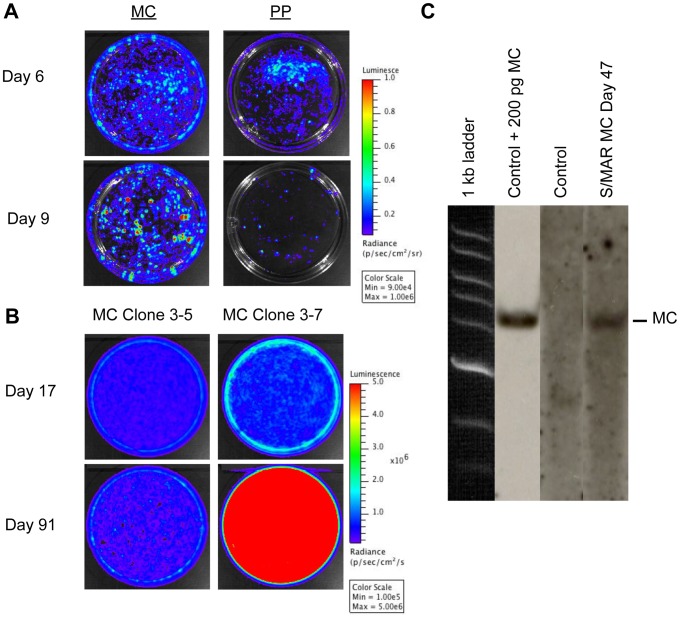
S/MAR MCs can label cells in culture for extended periods of time and remain episomal. A) pCMV-Luc2-S/MAR PP and MC constructs were transfected into MDA-MB-231 cells, grown in the absence of antibiotic selection, and BLI was performed over the course of 9 days. On day 6, both MC and PP showed strong BLI signal within the cells. However, by day 9, the MC-labeled cells continued to display strong signal and the signal from PP-labeled cells began to disappear. On this day MC-labeled clones displaying strong luminescent signal were isolated and expanded. B) Two clones (3–5 and 3–7) were cultured over the course of 91 days post-transfection and continued to be imaged. Both clones continued to show luminescent signal over the entire 3-month period. C) Southern blot analysis was performed on total DNA isolated from control cells, from control cells spiked with 200 pg of S/MAR MC, and from an S/MAR MC clonal population (clone 3-7) 47 days after transfection. Total DNA (40 µg) was digested with a single cutting enzyme and probed with a Luc2 probe. A single band at the correct size (4.5 kb) was detectable only in the lanes with control DNA spiked with the original construct (lane 1) and the S/MAR MC clone (lane 3), confirming the episomal nature of the construct.

Our first experimental objective was to establish the ability of our S/MAR MCs to label cultured cells with RGs for extended periods of time. MDA-MB-231 breast cancer cells were transfected with either PP or MC, grown without antibiotic selection, and imaged at both day 6 and 9 after transfection (Figure 2A). Unlike S/MAR PPs, S/MAR MCs do not require antibiotic selection to become established as replicating episomes. Therefore we expect that luciferase activity will be lost over time using S/MAR PPs but better maintained with S/MAR MCs. Cells were transfected with equal mass of PP and MC and therefore due to the inherent differences in transfection efficiencies we focused our comparisons of relative Fluc levels over time to changes within rather than between PP and MC cell populations. On day 6, both PP and MC showed strong bioluminescent signal. In contrast, at day 9, after several days of continued cell growth, the MC signal began to show foci of strong luminescent signal, whereas the PP signal began to disappear (Figure 2A). At this point individual S/MAR MC cell colonies that displayed high levels of bioluminescent signal were isolated and expanded to generate clonal cell populations. Several of these clones (clone 2-1, 3-5, and 3-7) were maintained in culture for extended periods of time (∼4 months) and serial BLI was performed (Figure 2B). As seen in Figure 2B, each clone continued to display bioluminescent signal for at least 3 months following transfection, indicating the ability to express RG in cells with S/MAR MCs for extended periods of time.

We next isolated total DNA from both control cells and one of our S/MAR MC clones (clone 3-7) and determined whether the construct existed as an episome by performing Southern blot analysis. After digesting total DNA with a single restriction enzyme (EcoRI) to linearize the MC and hybridizing with a Luc2 probe, we were able to detect a single band in our S/MAR MC clonal population at day 47 post-transfection (Figure 2C). Importantly this band showed up at the correct size for our S/MAR MC construct (4.5 kb), as was also shown for control DNA spiked with our S/MAR MC construct. No band was detectable in DNA from control cells (Figure 2C). Therefore, similar to previous studies [14]–[16], this confirms that our S/MAR MC construct exists as an episome and can replicate autonomously as cells divide.

**Figure 3:**
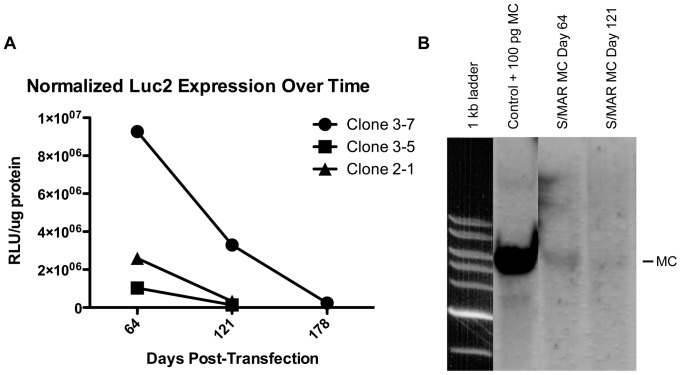
Luciferase activity and S/MAR MCs are slowly lost over time in labeled cell populations. A) Normalized luciferase expression was measured at both day 64 and 121 post-transfection in three S/MAR MC clonal populations. All clones showed a trend (p = 0.18) towards decreased normalized luciferase expression over long periods of time in culture. S/MAR clone 3-7 was cultured up to day 178 after transfection and continued to show a slow loss of luciferase activity. B) The decrease in luciferase expression corresponded to a decrease in Luc2-S/MAR MC as shown via Southern blot analysis. A single band was seen in both control DNA spiked with 100 pg of S/MAR MC and DNA from S/MAR MC clone 3-7 at day 64 post-transfection. However, a band is barely discernable at day 121 from S/MAR MC clone 3-7, indicating a slow loss of S/MAR MC over time.

**Both RG expression and episomes are slowly lost in S/MAR MC labeled cells** While we detected luminescent signal in the culture dish over time, the results in Figure 2B do not reflect differences in the number of cells in each dish at the time of imaging. Therefore, we performed a luminometer assay and protein assay on lysates from S/MAR MC clonal populations to measure Fluc activity (relative light units; RLU) normalized to protein content (µg) (Figure 3A). Comparing results at days 64 and 121 post-transfection across the three S/MAR MC clones we noted a trend (p = 0.18) towards decreased normalized Fluc activity (64% decrease for clone 3-7, 86% decrease for clone 3-5, and 87% decrease for clone 2-1), signifying a slow loss of Fluc activity over time. For S/MAR clone 3-7, which showed the highest Fluc activity of all clones and was cultured for the longest period of time, Fluc activity continued to decline up to day 178 post-transfection (97% decrease compared to day 64).

We then wanted to investigate whether this slow loss in Fluc activity corresponded to a decrease in S/MAR MC episomal content as assessed by Southern blot (Figure 3B). At day 64, as at day 47 in Figure 2C, we saw a clear single band indicating a significant number of episomes in this clonal population (clone 3-7). In contrast, at day 121 a band was barely discernible demonstrating that the S/MAR MC was slowly lost over time. Therefore, our results show that over long periods of time in culture labeling of cells with S/MAR MCs driven by the CMV promoter, results in a decreasing percentile of MC positive cells over time, and appears to be intrinsically reversible.

### S/MAR MC labeled cells can be implanted into small animals and cell proliferation can be monitored over time with non-invasive imaging

**Figure 4:**
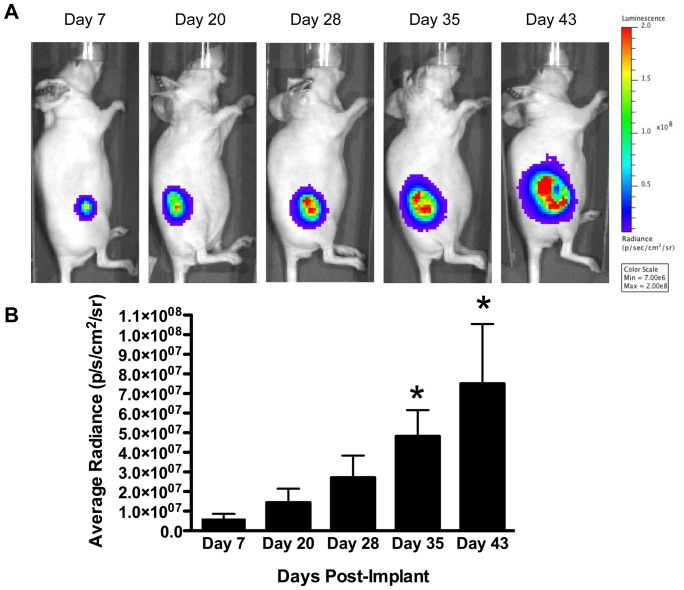
Proliferation of S/MAR MC labeled cells can be monitored over time in living subjects. A) S/MAR MC labeled breast cancer cells were implanted into the right flank of Nu/Nu mice and bioluminescence imaging (BLI) was performed over time. As tumors developed more luminescent signal was noted. B) This observation was confirmed by performing region of interest analysis over the tumor and measuring average radiance (p/s/cm^2^/sr) at days 7, 20, 28, 35 and 43 post-implantation. Significantly higher BLI signal (n = 5; * p<0.05) was noted at days 35 and 43 post-implantation compared to day 7. Error bars are S.E.M..

To show that S/MAR MCs can be used as a platform for tracking proliferating cells *in vivo* we implanted 1.5×10^6^ MDA-MB-231 Luc2-S/MAR MC labeled cells (clone 3-7; day 61 post-transfection) into the right flank of 6 week old female Nu/Nu mice and performed BLI over time (Figure 4 and Figure S1). As shown in Figure 4A, as tumors developed we saw an increase in the bioluminescent signal in the flank of these animals. We confirmed this observation by quantifying the amount of bioluminescent signal emanating from tumors. To do this, we performed ROI analysis over tumor sites and detected significant increases in average radiance at days 35 and 43 post-implantation compared to day 7. (Figure 4B). Therefore, as cells divided *in vivo* the S/MAR MCs continued to replicate, verifying that these constructs can be used to perform RG imaging of dividing cells in living subjects.

## Discussion

Many cell-based therapies are rapidly being developed for the treatment of a variety of devastating diseases. Direct imaging of cell fate has tremendous potential to improve the ability to assess therapeutic efficacy in individual patients. However, safety concerns regarding integrative technologies needed to genetically modify cells often represents a significant regulatory bottleneck that limits widespread adoption of RG technology into cell therapy trials. In this study, we showed that S/MAR MCs can be used to label cells in culture with imaging RGs over extended periods of time and that these constructs remain episomal within the cells. Unexpectedly but importantly, we also show evidence that with our S/MAR MC constructs RG expression is slowly lost over extended periods of time in culture and that this corresponds to a slow loss of S/MAR MCs. Lastly, we demonstrated the ability to implant cells labeled in culture into living subjects and to monitor *in vivo* cell proliferation and viability over extended periods of time (>40 days).

Two main cellular imaging approaches have been established. The first involves the labeling of cultured cells with imaging small molecules or nanoparticles followed by imaging of implanted cells *in vivo*. One of the main drawbacks of this technique are that as cells divide the imaging labels get diluted, making it difficult to track cells over extended periods of time and to quantify cell numbers. Furthermore, if a cell dies the label will still persist for some time and so false-positive cell imaging results are likely. The second method involves labeling of cultured cells with imaging RGs followed again by imaging of implanted cells in living subjects. The RG either intrinsically produces imaging signal such as fluorescent proteins [4] and some MRI reporter genes [21], [22], or expresses a protein that traps a systemically administered reporter probe (RP) such as BLI or PET RG/RP systems [5], [6] [8]. Importantly, no dilution of imaging signal is seen during cell division since each daughter cell receives a new copy of the RG, and critically, dead cells also no longer express the RG, allowing cell viability to be assessed. This makes RGs the ideal cell tracking technology, however until now the main drawback of RGs is the need to genetically modify the cells one wants to track.

Traditionally, cells that stably express RGs are modified using either viruses (e.g., lentiviruses) that readily, but randomly, integrate their genetic content into the genome [2], [23], or with plasmids followed by antibiotic selection [10], which also results in random integration into the genome. Dependent on where these integrations occur, these technologies can ultimately cause deregulation of endogenous genes. This may have important consequences on both the normal biological function of the cells [24], [25], or more importantly may activate nearby proto-oncogenes, transforming the therapeutic cell one wants to label into a malignant cell [11], [12]. Hence, if possible, these random integration technologies should be avoided and alternative vector platforms should be explored. In two very recent studies, site-directed integration of RGs into stem cells using either phiC31 integrases [26] or zinc-finger nucleases [27] has been explored as an alternative to random integrative technologies. These are exciting and promising ways to avoid critical sites within the genome associated with proto-oncogene activation and insert foreign genetic material into so-called genomic “safe harbours”. However, one caveat to this strategy is that this still requires genetic modification of the genome. Considering that the latest evidence (ENCODE project) ascribes approximately 80% of the genome with transcribed functional elements, both coding and non-coding RNAs, and 99% of the genome lies within 1.7 kb of a biochemical event [28], we believe an episomal technology that avoids integration altogether will be preferred from both functional and safety perspectives.

There are 3 examples of replicating episomal vector systems used to modify cells [29]. These include human artificial chromosomes (HACs), vectors composed of viral replication origins and trans-activating factors, and S/MAR vectors. The former two vector classes have their drawbacks. The major limitations of HACs relate to complexity of production and the difficulty to efficiently deliver these large vectors into target cells [29]. Alternatively, all known viral-based vectors rely on the expression of proteins known to induce cellular transformation, such as Epstein-Barr virus nuclear antigen 1 (EBNA-1) [30], and so it is unlikely that this vector class will have any clinical utility. In contrast, S/MAR MCs are easy to construct, can be readily introduced into cells, and exploit human chromosomal elements to induce replication and maintenance in progeny cells. Therefore, of the three vector platforms described to date, S/MAR vectors are arguably the most promising in terms of ease of use, safety, and clinical translatability.

The first S/MAR vector to be described, called pEPI-1, was a plasmid containing the hIFN-ß S/MAR and the SV40 origin of replication (ORI) [13]. This vector was shown to maintain mitotic stability by binding to matrix proteins such as SAF-A (also known as hnRNP-U)[31] and replicate episomally once-per-cell-cycle in a semiconservative fashion [13], [32]. Later it was shown that constructs containing a transcription unit linked to a downstream S/MAR (i.e. without an SV40 ORI) was sufficient for maintenance as an episome [33]. Unfortunately, these first generation S/MAR plasmids had two main drawbacks. First, the requirement for antibiotic selection to establish these vectors as replicating episomes violates regulatory “plasmids free of antibiotic resistance genes” (pFAR) principles [15], [34]. In addition, further investigation of S/MAR plasmids revealed that in some clonal cell populations these vectors can eventually integrate into the genome after long periods of time in culture, and therefore would not be pursued clinically [14]. In recent years this has led to removal of prokaryotic components and development of S/MAR MCs [14]–[16]. Compared to S/MAR plasmids, S/MAR MCs have several important safety advantages related to their eventual translation into humans; they do not require antibiotic selection to become established as episomes and also have a greater tendency to resist integration [14], [15]. Importantly, we have demonstrated in this study that S/MAR MCs can be used as a vector platform to label cells with RGs and track the proliferation and viability of transplanted cells in living subjects.

Recently, Argyros et al demonstrated the ability to label cancer cells with S/MAR “plasmids” for the purposes of developing imageable tumor models without the need to modify the cancer cell's genome [35]. In their study, the S/MAR plasmids were driven by the mammalian Ubiquitin C promoter, expressed firefly luciferase, and contained the hIFN-ß S/MAR. Two different types of cancer cell types were labeled and the ability to monitor cancer progression in mice with bioluminescence imaging was demonstrated. Our work supports the notion of using S/MAR vectors for monitoring tumor development in mice without the need to modify the cancer cell genome. We observed differences in the absolute amount of luciferase signal in cells transfected with either S/MAR PPs and S/MAR MCs (Figure 2A). Several reasons can explain these differences. Firstly, we transfected cells with equal mass of both PP and MC, therefore a greater number of transcription units were transfected using S/MAR MCs. Another partial explanation could be that without selection pressure S/MAR MCs are known to replicate whereas S/MAR PPs do not [14]. Another reason could be that there could be slightly more cells in the MC plate than the PP plate, however we did not see any obvious qualitative differences in cell number. Due to the different sizes of the constructs it would be difficult to assess absolute transfection efficiency without including another reporter within the construct itself. We did not pursue this strategy since it has been described previously [14], [15], and in our own experiences, that S/MAR constructs have a maximum cloning capacity, and therefore we wanted to minimize the overall size of our constructs. Critically, we believe S/MAR MCs will have significant advantages over S/MAR plasmids as we move towards our intended purpose: translation of these vectors for tracking therapeutic cells in patients with clinically relevant RGs. Safety will be of utmost importance for translation and as described above, S/MAR MCs have clear advantages over S/MAR plasmids regarding safety concerns.

Another advantage of our particular MC construct is that while our initial goal was to label cells indefinitely as has been previously described with other MC constructs [14]–[16], [35], serendipitously across several clonal cell populations we saw a consistent slow drift towards lower Fluc activity (Figure 3A) and S/MAR MC content over time (Figure 3B). To explain these findings, it has been previously shown that the ability of S/MAR vectors to replicate is dependent on ongoing transcription into the S/MAR motif [36]. Therefore, our theory for the loss of RG expression and vector over time relates to the selective use of the CMV promoter (pCMV) in our MC constructs. Studies have shown that loss of gene expression due to silencing is promoter-dependent [37], and that the CMV promoter is particularly prone to CpG methylation-based silencing both *in vitro* and *in vivo* [16], [38], [39]. In our case, the silencing of pCMV would result in a gradual inhibition of RG transcription, inhibition of the ability of the MC to replicate, and as those cells continue to divide the non-replicative MC would eventually be lost. This provides an exciting way to reversibly modify cells with RGs after a prolonged imaging window (months), and is even safer and may have many more specific applications in the clinic compared to a system that modifies cells indefinitely. Future work will focus on development of MC constructs that utilize mammalian promoters, such as the Ubiquitin C promoter [35], that do not easily get silenced to cover applications that require longer cell imaging windows and, as has been recently explored [40], the use of inducible promoters that can be turned off once the appropriate imaging window has been achieved.

One of the limitations of our current generation of S/MAR MCs is the use of the 2.2 kb hIFN-ß S/MAR motif. In an detailed study by Broll and colleagues using MC constructs containing this S/MAR, a few clonal populations expanded after FAC sorting showed evidence of eventual integration after long periods in culture (5 months) [15]. Of note though, this same study described a shorter S/MAR motif (733 bp) called M18, identified after *in vivo* recombination in one of their clonal populations, that when incorporated into MC constructs showed largely improved cloning capacity and stability [15]. Another limitation of S/MAR vectors in general is the low establishment rate within cells; with S/MAR MCs it is estimated that the final rate of establishment is less than 5 percent [15]. This appears to be regulated at the epigenetic level, since treatment of cells prior to transfection with histone deactylase (HDAC) inhibitors, such as butyrate, to open up the cell's chromatin structure can improve the establishment rate [15]. Continued work exploring the effects of other HDAC inhibitors or other epigenetic factors that can be modulated to improve establishment rates are warranted.

To our knowledge, this is the first work exploring the use of replicating, self-limited episomal MCs to monitor cell proliferation and viability in living subjects. S/MAR MCs are a promising non-integrative, replicative vector platform that avoids the safety issues of integrative technologies but provides an imaging window (>3 months) that would allow clinicians to monitor cell fate early in the treatment process and, if needed, intervene in a timely fashion. This work was supported by

## Supporting Information

Figure S1 **Proliferation of S/MAR MC labeled cells can be monitored over time in living subjects.** S/MAR MC labeled breast cancer cells were implanted into the right flank of Nu/Nu mice and bioluminescence imaging (BLI) was performed over time. As tumors developed more luminescent signal was noted.
(DOCX)
