# Supplementary figures and images for: Development and Validation of Non-Integrative, Self-Limited, and Replicating Minicircles for Safe Reporter Gene Imaging of Cell-Based Therapies
Source: PLoS One. 2013 Aug 28;8(8):e73138. doi: 10.1371/journal.pone.0073138 (PMC3756008; doi:10.1371/journal.pone.0073138)

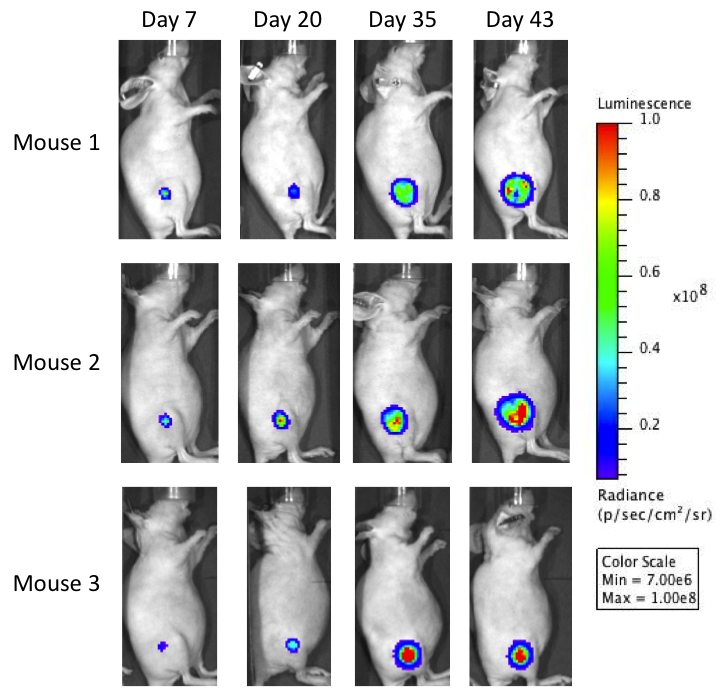

Supplement: Figure S1 — Proliferation of S/MAR MC labeled cells can be monitored over time in living subjects. S/MAR MC labeled breast cancer cells were implanted into the right flank of Nu/Nu mice and bioluminescence imaging (BLI) was performed over time. As tumors developed more luminescent signal was noted. (DOCX) [file pone.0073138.s001.docx]
